# Comprehensive Exploration of Three Pistachio Varieties: Antidiabetic, Anticholinergic, Antioxidant Properties, Phytochemical Profile by LC–MS/MS, and Molecular Docking

**DOI:** 10.1002/fsn3.71656

**Published:** 2026-03-23

**Authors:** Ebubekir İzol, Büşra Beltekin, Münire Turhan, Şirvan Karakoç, Adem Necip, Mustafa Abdullah Yılmaz, Gökhan Zengin, Oğuz Çakır

**Affiliations:** ^1^ Bee and Natural Products R&D and P&D Application and Research Center, Bingöl University Bingöl Turkey; ^2^ Department of Bee and Bee Products, Institute of Science and Technology, Bingöl University Bingöl Turkey; ^3^ Faculty of Agriculture, Vocational School of Food, Agriculture and Livestock, Bingöl University Bingöl Turkey; ^4^ Department of Animal Nutrition and Nutritional Diseases, Faculty of Veterinary Medicine, Fırat University Elazığ Turkey; ^5^ Department of Pharmacy Services, Vocational School of Health Services, Harran University Şanlıurfa Turkey; ^6^ Department of Analytical Chemistry, Faculty of Pharmacy, Dicle University Diyarbakır Turkey; ^7^ Department of Biology, Science Faculty, Selcuk University Konya Turkey; ^8^ Department of Nutrition and Dietetics, Atatürk Faculty of Health Sciences, Dicle University Diyarbakır Turkey

**Keywords:** antioxidant activity, enzyme inhibitory activity, molecular docking, phytochemicals, *Pistacia vera*

## Abstract

Pistachios ( *Pistacia vera* L.) are a significant food that is added to many foods and also consumed as a snack. The kernel part is consumed very much. In this study, 53 phytochemical constituents of three pistachio varieties (Halebi, Kırmızı, and Uzun) were profiled by LC–MS/MS, and their antioxidant capacity (total phenolic and flavonoid content, ABTS, DPPH, CUPRAC, FRAP, MCA, and phosphomolybdenum assays), antidiabetic activity (α‐amylase and α‐glucosidase inhibition), anticholinergic activity (AChE and BChE inhibition), and wound healing potential (tyrosinase inhibition) were evaluated. Additionally, molecular docking was used to examine the binding interactions of the major phytochemicals with each enzyme. The major phytochemical ingredients were tannic acid (24.005 mg/g), catechin (11.284 mg/g), protocatechuic acid (6.202 mg/g), gallic acid (5.717 mg/g), epicatechin gallate (1.686 mg/g), and epigallocatechin gallate (1.139 mg). The highest antioxidant properties were obtained in the Uzun variety in all assays. Antioxidant properties were high in all three varieties. While wound healing potential was high across all three varieties (30–43 mg GALAE/g), their anticholinesterase (2.06–2.54 mg GALAE/g) and antidiabetic activities (0.64–2.94 mmol ACAE/g) were comparatively low. Major bioactive phytochemicals were discovered to have highly favorable docking scores. This study determined that three types of pistachios contain significant bioactive compounds and are a very nutritious food due to the diversity of their biological activities.

## Graphical abstract

Phytochemical profiles, biological activities, and molecular docking studies of pistachio varieties.

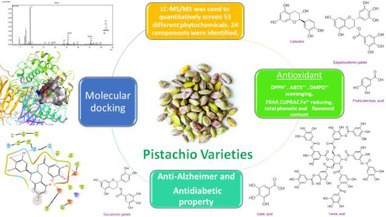

## 1 Introduction

*Pistacia vera* L., a member of the Anacardiaceae family, is a popular snack that can be eaten fresh, roasted, or salted. It has a wonderful taste, high nutritional content, and a composition that promotes health (Noguera‐Artiaga et al. 2018; Carbonell‐Barrachina et al. 2015). The edible seed in the ripe fruit is called a “pistachio,” and a pistachio nut consists of a fleshy shell (epicarp and mesocarp) that surrounds the nut shell and encloses the nut flesh (Gok et al. 2022; Grace et al. 2016). As an ingredient in fermented meats, ice cream, milk, bread, sauces, sausages, biscuits, and pudding, pistachios are widely used in the food sector (Mertdinç et al. 2023; Everest 2021; Sonmezdag et al. 2018; Tomaino et al. 2010). Today, Italy, Iran, Syria, Greece, the United States, Spain, and Türkiye all grow pistachios as a major agricultural commodity (Rodríguez‐Bencomo et al. 2015). Pistachios are mainly grown in the southeastern region of Türkiye. The most commonly grown pistachio variety in this region is “Uzun” because of its uniform green kernels and distinctive aroma (Sonmezdag et al. 2018). This type of pistachio is delicious and small‐grained, and its requirement is lower than “Halebi.” The other type of pistachio grown in Türkiye is “Halebi.” With its small, tasty fruits, “Halebi” is particularly recommended for rare regions, as it is the earliest flowering variety of all standard plants, has low temperatures, and is widely cultivated in Gaziantep Province (Karaca and Nizamoglu 1995). Another variety is “Tekin.”

The fruits and seeds of *Pistacia vera* L. have been reported to have positive effects in reducing the main risk factors for cardiovascular diseases, abdominal discomfort, abscesses, gynecological complaints, bruises, itching and sores, breast diseases, endothelial dysfunction, and amenorrhea. Dysentery, blood pressure, inflammation, skin diseases, and oxidative status (Bisignano et al. 2013; Boukeloua et al. 2016; Liu 2004; Noguera‐Artiaga et al. 2018). These bioactive properties draw attention to the components of pistachio. Beyond their economic value, pistachios are rich in phenolic compounds and are among the 50 foods with the highest antioxidant properties. They may therefore be regarded as a special kind of functional food (Halvorsen et al. 2006). Prior research has shown that pistachios' shell, stem, blossoms, leaves, and oil all contain trace levels of phenolic chemicals in addition to their kernels (Yilmaz, Cakir, and Yener 2023; Tomaino et al. 2010; Martorana et al. 2013; Goli et al. 2005). Phenolic substances, such as flavonols, flavanones, isoflavones, flavan‐3‐ols, anthocyanins, proanthocyanidins, phenolic acids, and stilbenes, are widely known for their strong antioxidant activity, anti‐chemotherapy, cardioprotective, and vasoprotective qualities (Fidan et al. 2021; Shukitt‐Hale et al. 2006; Tomaino et al. 2010; Yeniçeri et al. 2024; Yiğitkan et al. 2021).

By eliminating oxidative stress and free radicals from the body, antioxidants are at the forefront of the treatment of numerous illnesses (İzol, Yılmaz, and Gülçin 2025; Yurt et al. 2024; Çelik et al. 2024; Yilmaz et al. 2024). Enzyme inhibitors are also utilized in the production of medications and the treatment of certain serious illnesses (Yilmaz, Cakir, Izol, et al. 2023; Karageçili et al. 2023a; Bursal et al. 2021; Izol et al. 2021). It is known that the biological activity of foods is due to the phytochemicals they contain. LC–MS/MS is one of the most sensitive and reliable phytochemical methods (Yapıcı et al. 2025; Karageçili et al. 2023b). Molecular docking studies are used in many pharmacological research projects. They are actively used, particularly in identifying molecules with potential drug properties. The ligand conformations at the macromolecular target binding site are analyzed to determine an estimate of the receptor‐ligand binding free energy for each conformation. The ligand conformations generated at the macromolecular binding site were evaluated using the AutoDock scoring function, which estimates receptor–ligand binding free energy based on a weighted combination of intermolecular hydrogen bonding, van der Waals interactions, electrostatics, desolvation, and torsional entropy penalties, yielding the lowest‐energy pose as the predicted binding mode (Huey et al. 2007). The degrees of freedom of the ligand's torsion, translation, and rotation are sampled stochastically or deterministically using a conformational sampling (search) algorithm to determine the position with the most favorable binding free energy, after which the complex's binding affinity is determined. One important computational technique for examining how chemicals interact with proteins is molecular docking, which predicts the binding mode and affinity of small molecules in the active sites of protein targets (İzol 2025; İzol, Turhan, Yapıcı, et al. 2025).

In this study, the comprehensive antioxidant (with eight different assays), antidiabetic, anticholinergic, and wound‐healing properties of three different *Pistacia vera* varieties (Uzun, Halebi and Tekin) were determined. In addition, 53 phytochemicals were screened by LC–MS/MS to identify the bioactive components. Molecular docking calculations were used to predict the intermolecular interactions and affinities of epicatechin gallate, catechin, epigallocatechin gallate, gallic acid and isoquercitrin ligands against 6GXV, 2Y9X, 5NN8, 6EQP and 4EY7 proteins. The effects and responses of these compounds in relation to human metabolism were then assessed using ADME/T calculations.

## 2 Material and Method

### 2.1 Chemicals

Chemicals and standards used in enzyme inhibition studies, antioxidant and LC–MS/MS analyses were obtained from Sigma‐Aldrich (Steinheim, Germany). The chemicals were of analytical purity.

### 2.2 Supply of Samples

*Pistacia vera* L. samples are supplied from the Republic of Türkiye Ministry of Agriculture and Forestry Pistachio Research Institute, Gaziantep. The pistachio types used in this research are Uzun, Tekin, and Halebi (Yilmaz, Cakir, Izol, et al. 2023).

### 2.3 Preparation of the Samples

The pistachio samples were left to dry in the dark and at room temperature. Ten grams of dried materials were macerated independently with 50 mL of methanol for 24 h three times at room temperature in order to observe the extracts. Before LC–MS/MS and bioactivity assays, crude extracts were prepared using a rotary evaporator (under vacuum; 45°C) to remove solvents. They were then kept between 0°C and 8°C in the dark.

### 2.4 Identification of Phytochemicals Qualitatively and Quantitatively by LC–MS/MS

The phytochemicals in the ethanolic, methanolic, and water extracts of pistachio samples were assessed both qualitatively and quantitatively using a previously created and approved LC–MS/MS method (Yilmaz 2020; İzol, Turhan, Yılmaz, et al. 2025). 53 phytochemicals were measured using a Shimadzu‐Nexera type ultrahigh performance liquid chromatograph (UHPLC) and a triple quadrupole mass spectrometer. A column oven (CTO‐10ASvp type), autosampler (SIL‐30AC model), binary pumps (LC‐30ce model), and degasser were all included with the reversed‐phase UHPLC (DGU‐20 A3R model). The Agilent Poroshell 120 EC‐C18 model (150 mm × 2.1 mm × 2.7 m) reversed‐phase analytical column was used to perform the chromatographic separation. The temperature of the column was fixed at 40°C. Eluents A (water + 5 mM ammonium formate + 0.1% formic acid) and B (methanol + 5 mM ammonium formate + 0.1% formic acid) made up the elution gradient. A gradient elution profile of 20%–100% B (0–25 min), 100% B (25–35 min), and 20% B (35–45 min) was also employed. Additionally, the injection volume and solvent flow rate were set at 5 μL and 0.5 mL/min, respectively. A Shimadzu brand LCMS‐8040 tandem mass spectrometer with an electrospray ionization (ESI) source that could be used in both positive and negative ionization modes was employed for the mass spectrometric detection. The LabSolutions software from Shimadzu was used to collect and process the LC–MS/MS data. The phytochemicals were quantified using the multiple reaction monitoring, or MRM, technique. Based on the screening of specific precursor phytochemical‐to‐fragment ion transitions, the MRM method was developed to identify and detect phytochemical molecules exclusively. In order to attain optimal photochemical fragmentation and optimum transmission of the desired product ions, the collision energies (CE) were modified. The MS operating parameters were as follows: 350°C for the interface, 400°C for the heat block, and 250°C for the DL; 15 L/min of nitrogen drying gas flow; and 3 L/min of nitrogen nebulizing gas flow (Erzincan et al. 2025).

### 2.5 Total Amounts of Flavonoids and Phenols

The Folin–Ciocalteu and AlCl_3_ tests were used to assess the total phenolic and flavonoid contents, respectively (Karageçili et al. 2023b; Onder et al. 2022). The results of each assay were expressed as gallic acid equivalents (mg GAEs/g extract) and rutin equivalents (mg REs/g extract). The total phenolic content was determined using the method outlined in the literature (İzol 2025; Slinkard and Singleton 1977), with a few minor adjustments. The sample solution (1 mg/mL; 0.25 mL) was mixed with the diluted Folin–Ciocalteu reagent (1 mL, 1:9, v/v) and swirled rapidly. 0.75 mL of 1% Na_2_CO_3_ solution was added after 3 min of incubation, and the sample absorbance at 760 nm was evaluated 2 h later at room temperature.

According to Bouagnon et al. (2024), milligrams of gallic acid equivalents (mg GAE/g extract) were used to measure the total phenolic content. The total flavonoid content was determined using the AlCl_3_ technique. To put it briefly, the sample solution (1 mg/mL; 1 mL) was mixed with the equivalent volume of aluminum trichloride (2%) in methanol. Similarly, a blank was made by mixing 1 mL of methanol with 1 mL of sample solution without AlCl_3_. The absorbances of the sample and blank were measured at 415 nm following a 10‐min incubation period at room temperature. The sample's absorbance was subtracted from the blank's. Using rutin as the reference standard, the total flavonoid content was measured in milligrams of rutin equivalents (mg RE/g extract) (Yıldız et al. 2024).

### 2.6 Antioxidant Activity Assays

The DPPH (1,1‐diphenyl‐2‐picrylhydrazyl) radical and the ABTS ((2,2′‐azinobis(3‐ethylbenzothiazoline)‐6‐sulphonic acid)) radical cation were assessed for their scavenging properties using recognized methods. Trolox equivalents (TEs/g extract) were used to express the results. As previously reported, the FRAP (ferric ion reducing antioxidant power) and CUPRAC (cupric ion reducing antioxidant power) methodologies were used to evaluate the extracts' reducing power. Trolox equivalents (TEs/g extract) were used to represent the results.

The method used to determine total antioxidant capacity included phosphomolybdenum. EDTA equivalents (EDTAEs/g extract) were used to express the results of the assessment of the extracts' ability to chelate metals from ferrous ions. Other publications have already described the methods for measuring antioxidant activity (Yilmaz, Cakir, Izol, et al. 2023; Zengin et al. 2017).

The DPPH radical scavenging experiment was conducted by mixing 1 mL of the sample solution (1 mg/mL) with 4 mL of a 0.004% methanol solution of DPPH. Before measuring the sample's absorbance at 517 nm, it was incubated for 30 min at room temperature in the dark. The DPPH radical scavenging activity was expressed using trolox equivalents (mg TE/g extract).

To summarize the ABTS radical scavenging test, 2.45 mM potassium persulfate was added to 7 mM ABTS solution, and the mixture was allowed to sit at room temperature in the dark for 12–16 h. Before the test, methanol was added to the ABTS solution to dilute it, and the absorbance at 734 nm was 0.700 ± 0.02. The sample solution (1 mg/mL; 1 mL) was mixed with the ABTS solution (2 mL). Following a half‐hour incubation period at room temperature, the absorbance at 734 nm was measured. The amount of ABTS radical scavenging activity was measured using a millimole of trolox equivalents (mmolTE/g extract).

For the CUPRAC activity test, a premixed reaction mixture including CuCl_2_ (1 mL, 10 mM), neocuproine (1 mL, 7.5 mM), and NH_4_Ac buffer (1 mL, 1 M, pH 7.0) was mixed with the sample solution (1 mg/mL; 0.5 mL). Similarly, 0.5 mL of the sample solution (without CuCl_2_) was combined with 3 mL of the premixed reaction mixture to create a blank. After a 30‐min incubation period at room temperature, the absorbances of the sample and blank were then measured at 450 nm. The sample's absorbance was subtracted from the blank's.

A premixed FRAP reagent (2 mL) consisting of ferric chloride (20 mM), 2,4,6‐tris(2‐pyridyl)‐S‐triazine (TPTZ) (10 mM) in 40 mM HCl, and acetate buffer (0.3 M, pH 3.6) was added to the sample solution (1 mg/mL; 0.1 mL) in order to conduct the FRAP activity experiment. The FRAP reagent and sample solution were mixed in a 10:1:1 (v/v/v) ratio. The sample absorbance was measured at 593 nm and recorded following a 30‐min incubation period at room temperature.

The sample solution (1 mg/mL; 0.3 mL) was mixed with 3 mL of the reagent solution (0.6 M sulfuric acid, 28 mM sodium phosphate, and 4 mM ammonium molybdate) for the phosphomolybdenum method. After 90 min of incubation at 95°C, the sample absorbance was measured at 695 nm.

For the metal chelating activity test, sample solution (1 mg/mL; 2 mL) was combined with FeCl_2_ solution (0.05 mL, 2 mM). The procedure began with the addition of 0.2 mL of 5 mM ferrozine. A blank was made by following the same steps: combining 2 mL of sample solution with 0.05 mL of FeCl_2_ solution (2 mM) and 0.2 mL of water (without ferrozine). After a 10‐min incubation time at room temperature (25°C), the absorbances of the sample and blank were then measured at 562 nm. The sample's absorbance was subtracted from the blank's. The metal chelating activity was measured in milligrams of EDTA (disodium edetate) equivalents (mg EDTAE/g extract) (Yilmaz et al. 2024).

### 2.7 Enzyme Inhibitory Activity Assays

To ascertain the inhibitory effects of extracts on a variety of enzymes, such as tyrosinase, α‐amylase, α‐glucosidase, butyrylcholinesterase (BChE), acetylcholinesterase (AChE), and α‐amylase, the current investigation used a tried‐and‐true methodology (Lobine et al. 2021). The standard compound equivalents utilized to express the data on the enzyme inhibitory activities were acarbose (mmol ACAEs/g extract) for α‐amylase and α‐glucosidase, galathamine (mg GALAEs/g extract) for AChE and BChE, and kojic acid (mg KAEs/g extract) for tyrosinase. Kojic acid as mg KAEs/g extract for α‐amylase, and galathamine as mg GALAEs/g extract for AChE and BChE were the standard chemical equivalents used to describe the enzyme inhibitory activity data.

The sample solution (1 mg/mL; 50 mL) was mixed with DTNB (5,5‐dithio‐bis (2‐nitrobenzoic) acid) (125 mL) and either AChE or BChE solution (25 mL) in tris–HCl buffer (pH 8.0) in a 96‐well microplate to conduct the ChE inhibitory activity assay. After that, the mixture was incubated at 25°C for 15 min. The reaction was then initiated by adding 25 mL of either acetylthiocholine iodide (ATCI) or butyrylthiocholine iodide (BTCl). Similarly, a blank was made by substituting the sample solution for the enzyme (AChE or BChE) solution in each reaction reagent. The absorbances of the sample and the blank were measured at 405 nm following 10 min at 25°C. The galanthamine equivalents (mgGALAE/g) were used to quantify the cholinesterase inhibitory action. The absorbance of the sample was deducted from that of the blank in order to calculate the galanthamine equivalents (mgGALAE/g extract) of the cholinesterase inhibitory activity.

A 96‐well microplate was filled with tyrosinase solution (40 mL, Sigma), phosphate buffer (100 mL, pH 6.8), and sample solution (1 mg/mL; 25 mL). The mixture was then incubated for 15 min at 25°C. The reaction was then initiated by adding 40 mL of L‐DOPA. Similarly, the only reaction reagent to which the sample solution was added to produce a blank was the enzyme (tyrosinase) solution. The absorbances of the sample and the blank were measured at 492 nm following a 10‐min incubation period at 25°C. Tyrosinase inhibitory activity was expressed as mgKAE/g of extract (kojic acid equivalents) after the absorbance of the sample was subtracted from that of the blank. The absorbances of the blank and sample were measured at 630 nm. After subtracting the sample's absorbance from the blank's, the α‐amylase inhibitory activity was expressed in acarbose equivalents (mmol ACE/g extract).

In order to test the α‐glucosidase inhibitory activity, the sample solution (1 mg/mL; 50 mL) was combined with 50 mL of glutathione, 50 mL of α‐glucosidase solution in phosphate buffer (pH 6.8), and 50 mL of PNPG (4‐N‐trophenyl‐a‐D‐glucopyranoside) in a 96‐well microplate. After that, the mixture was incubated at 37°C for 15 min. Similarly, the sample solution was combined with all reaction reagents except the enzyme (α‐glucosidase) solution to form a blank. To stop the reaction, 50 mL of 0.2 M sodium carbonate was subsequently added. The absorbances of the blank and sample were measured at 400 nm. After subtracting the absorbance of the sample from the blank, the α‐glucosidase inhibitory activity was expressed using acarbose equivalents (mmol ACE/g extract) (Yilmaz et al. 2024).

### 2.8 Molecular Docking Studies

Docking is a crucial technique for identifying compounds with strong activity against biological materials. The crystal structures of Crystal structure of PPO3, a tyrosinase from Agaricus bisporus, in deoxy‐form that contains an additional unknown lectin‐like subunit, with inhibitor tropolone (PDB ID: 2Y9X, Method: X‐ray Diffraction, Resolution: 2.78 Å), Amylase in complex with acarbose (PDB ID: 6GXV, Method: X‐ray Diffraction, Resolution: 2.078 Å), Crystal structure of human lysosomal acid‐alpha‐glucosidase, GAA, in complex with acarbose (PDB ID: 5NN8, Method: X‐ray Diffraction, Resolution: 2.45 Å), Human butyrylcholinesterase in complex with ethopropazine (PDB ID: 6EQP, Method: X‐ray Diffraction, Resolution: 2.35 Å) and Crystal Structure of Recombinant Human Acetylcholinesterase in Complex with Donepezil (PDB ID: 4EY7, Method: X‐ray Diffraction, Resolution: 2.35 Å) were retrieved from the PDB database (http://www.rcsb.org/pdb). Schrödinger's Maestro molecular modeling platform was used to carry out molecular docking simulations. The LigPrep module is used to prepare the molecule once the protein has been prepared using the protein preparation module. Additionally, Glide ligand docking was used to dock prepared phytochemicals against the defined protein targets (Schrödinger Release 2022‐4 2022a, 2022b, 2022c). Ligands were prepared using the LigPrep module by applying energy minimization with the OPLS3e force field, generation of appropriate ionization states (pH 7.0 ± 0.5) using Epik, conversion to low‐energy 3D structures, and optimization of tautomers and stereochemistry prior to docking.

### 2.9 ADME Analysis

The Swiss ADME online web tool (http://www.swissadme.ch/) and Admetlab (https://admetmesh.scbdd.com/) were used to perform ADME analysis of catechin, epicatechin gallate, epigallocatechin gallate, gallic acid, and isoquercitrin. These compounds' canonical SMILES were created using ChemDraw, and physicochemical properties such as drug similarity, pharmacokinetics, TPSA, number of rotatable bonds, lipophilicity, and violations of Lipinski's five rules were predicted (Yildirim, Yasar, et al. 2025). ADME/T analysis was conducted to investigate the impact of the compounds under study on human metabolism.

### 2.10 Statistical Analysis

Experimental studies were carried out in three parallel runs. Mean and standard deviation (SD) values are shown with the results. One‐way analysis of variance (ANOVA) with SPSS v.14.0 was used to compare the differences between the various extracts, and Tukey's significant difference post hoc test with D 0.05 was used to confirm the results.

## 3 Results and Discussion

### 3.1 LC–MS/MS Analysis of *P. vera* Varieties

**FIGURE 1:**
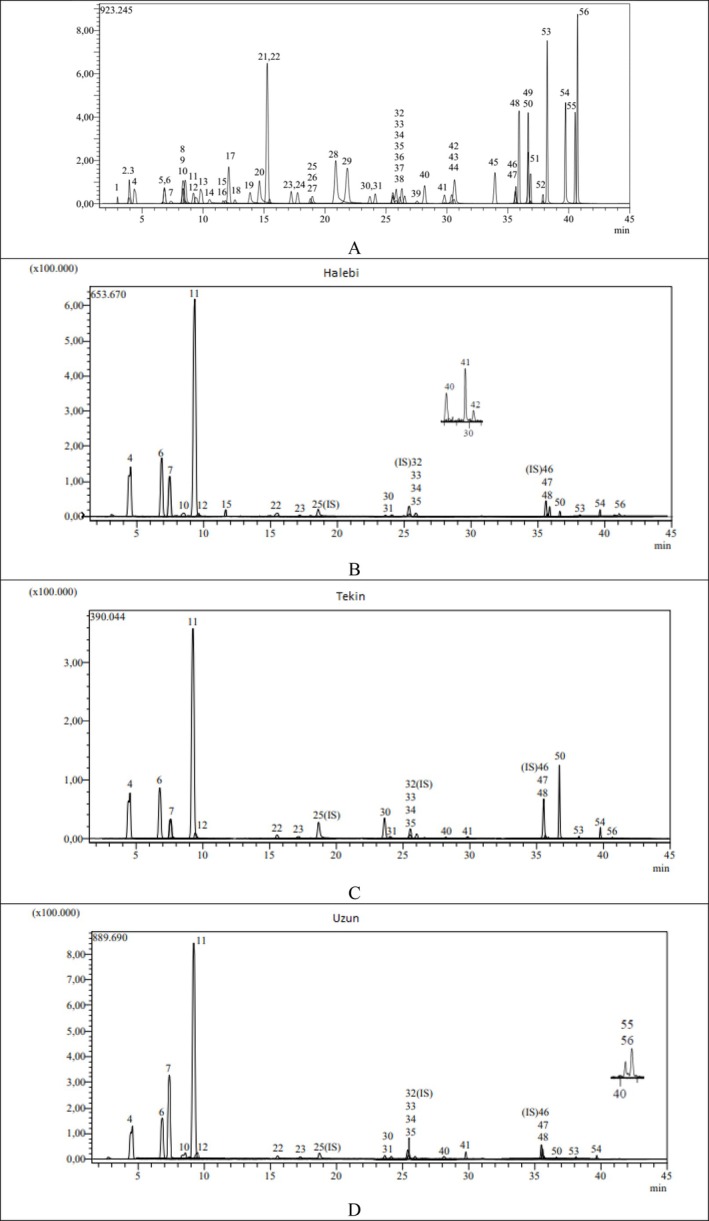
(A) LC–MS/MS chromatogram of the standards, (B) chromatogram of Halebi, (C) chromatogram of Tekin, and (D) chromatogram of Uzun.

Phytochemical analysis results of *P. vera* varieties are given in Table 1. Fifty‐three different phytochemicals and 3 different internal standards were used. Chromatograms of the standards and samples are presented in Figure 1.

**TABLE 1:** Phytochemical content results of *P. vera* varieties (Halebi, Tekin, Uzun) (mg analyte/g extract).

*N*	Phytochemical content	R.T.	M.I. (m/z)	F.I. (m/z)	Halebi	Tekin	Uzun
1	Quinic acid	3.0	190.8	93.0	—	—	—
2	Fumaric aid	3.9	115.2	40.9	—	—	—
3	Aconitic acid	4.0	172.8	129.0	—	—	—
4	Gallic acid	4.4	168.8	79.0	5.717	3.086	5.15
5	Epigallocatechin	6.7	304.8	219.0	—	—	—
6	Protocatechuic acid	6.8	152.8	108.0	6.202	3.359	6.003
7	Catechin	7.4	288.8	203.1	4.902	1.28	11.284
8	Gentisic acid	8.3	152.8	109.0	—	—	—
9	Chlorogenic acid	8.4	353.0	85.0	—	—	—
10	Protocatechuic aldehyde	8.5	137.2	92.0	0.036	—	0.042
11	Tannic acid	9.2	182.8	78.0	**17.054**	**9.756**	**24.005**
12	Epigallocatechin gallate	9.4	457.0	305.1	0.394	0.064	1.139
13	Cynarin	9.8	515.0	191.0	—	—	—
14	4‐OH Benzoic acid	10.5	137.2	65.0	—	—	—
15	Epicatechin	11.6	289.0	203.0	1.36	—	—
16	Vanilic acid	11.8	166.8	108.0	—	—	—
17	Caffeic acid	12.1	179.0	134.0	—	—	—
18	Syringic acid	12.6	196.8	166.9	—	—	—
19	Vanillin	13.9	153.1	125.0	—	—	—
20	Syringic aldehyde	14.6	181.0	151.1	—	—	—
21	Daidzin	15.2	417.1	199.0	—	—	—
22	Epicatechin gallate	15.5	441.0	289.0	1.323	0.515	1.686
23	Piceid	17.2	391.0	135/106.9	0.054	0.03	0.096
24	*p*‐Coumaric acid	17.8	163.0	93.0	—	—	—
25	Ferulic acid‐D3‐IS	18.8	196.2	152.1	N.A.	N.A.	N.A.
26	Ferulic acid	18.8	192.8	149.0	—	—	—
27	Sinapic acid	18.9	222.8	193.0	—	—	—
28	Coumarin	20.9	146.9	103.1	—	—	—
29	Salicylic acid	21.8	137.2	65.0	—	—	—
30	Cyranoside	23.7	447.0	284.0	0.129	1.078	0.178
31	Miquelianin	24.1	477.0	150.9	0.108	0.017	0.04
32	Rutin‐D3‐IS	25.5	612.2	304.1	N.A.	N.A.	N.A.
33	Rutin	25.6	608.9	301.0	0.169	0.162	0.118
34	Isoquercitrin	25.6	463.0	271.0	0.71	0.281	0.464
35	Hesperidin	25.8	611.2	449.0	0.049	0.046	0.038
36	*o*‐Coumaric acid	26.1	162.8	93.0	—	—	—
37	Genistin	26.3	431.0	239.0	—	—	—
38	Rosmarinic acid	26.6	359.0	197.0	—	—	—
39	Ellagic acid	27.6	301.0	284.0	—	—	—
40	Cosmosiin	28.2	431.0	269.0	0.017	0.04	0.025
41	Quercitrin	29.8	447.0	301.0	0.08	0.087	0.115
42	Astragalin	30.4	447.0	255.0	0.043	—	—
43	Nicotiflorin	30.6	592.9	255.0/284.0	—	—	—
44	Fisetin	30.6	285.0	163.0	—	—	—
45	Daidzein	34.0	253.0	223.0	—	—	—
46	Quercetin‐D3‐IS	35.6	304.0	275.9	N.A.	N.A.	N.A.
47	Quercetin	35.7	301.0	272.9	0.197	0.06	0.138
48	Naringenin	35.9	270.9	119.0	0.112	0.03	0.086
49	Hesperetin	36.7	301.0	136.0/286.0	—	—	—
50	Luteolin	36.7	284.8	151.0/175.0	0.038	0.283	0.018
51	Genistein	36.9	269.0	135.0	—	—	—
52	Kaempferol	37.9	285.0	239.0	—	—	—
53	Apigenin	38.2	268.8	151.0/149.0	0.003	0.007	0.002
54	Amentoflavone	39.7	537.0	417.0	0.038	0.04	0.023
55	Chrysin	40.5	252.8	145.0/119.0	—	—	0.003
56	Acacetin	40.7	283.0	239.0	0.007	0.007	0.005

*Note:* The bold letters indicate the highest concentration.

Abbreviations: —, not detected; D3, deuterium isotope 3; FI (m/z), fragment ions; IS, internal standard; M.I. (m/z), molecular ions of the standard analytes (m/z ratio); *N*, numbers; N.A., not applicable; R.T., retention time.

**FIGURE 2:**
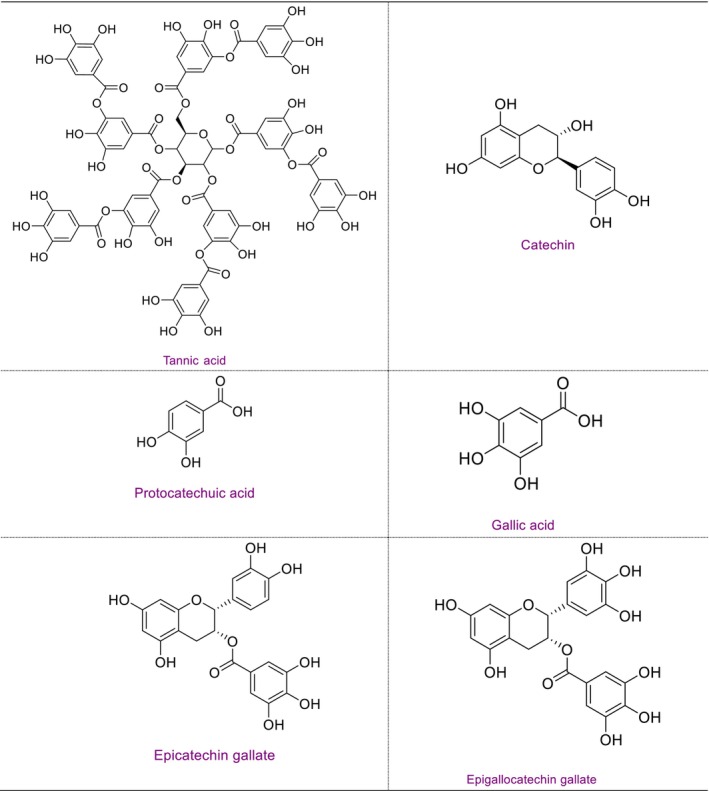
High concentrations of phytochemicals found in *P. vera* varieties.

In general, predominantly the same components were detected in the three varieties, but the concentrations were different. Tannic acid was found as the main component in all three varieties, and the highest concentration was found in the Uzun varieties. In general, the components with high concentrations were tannic acid > catechin > protocatechuic acid > gallic acid > epicatechin gallate > epigallocatechin gallate. The molecular structures of these phytochemicals are listed in Figure 2. The presence of different bioactive compounds indicates that regular consumption of these three types of pistachios as food has a positive effect on health.

In the study, which determined the polyphenol content of 11 varieties of pistachio nuts, 56 compounds were qualitatively and quantitatively determined using ultra‐high performance liquid chromatography‐mass spectrometry (UHPLC–MS). The most abundant compound in most cultivars was 3,4‐dihydroxybenzoic acid, and the second most abundant compound was vanillic acid hexoside. It was observed that there were differences in polyphenol content between varieties (Moreno‐Rojas et al. 2022). 11 pistachio varieties are different from 3 cultivars in this study. In addition, when compared with this study, it was determined that the varieties contained different phenolic compounds from each other. Gallic acid, epicatechin gallate, epigallocatechin gallate, epigallocatechin gallate, and catechin were detected in all varieties in the two studies. This result showed that these components were present in 14 different pistachio varieties, and their high concentrations indicate that these components can be isolated from pistachios. In 11 varieties, benzoic acid derivatives were detected, but not in the varieties in this study.

### 3.2 Antioxidant Activity Results

Total phenolic and flavonoid content and antioxidant activity results of ethanol extract of *P. vera* varieties are shown in Table 2.

**TABLE 2:** Total phenolic‐flavonoid contents, antioxidant, and enzyme inhibitory activities of *P. vera* varieties–EtOH extract.

Samples	Phenolic contents		Antioxidant activities					
	Total phenolic and flavonoid contents		Free radical scavenging activity		Total antioxidant capacity	Reducing power		
	TPC (mgGAE/g)	TFC (mgRE/g)	DPPH (mgTE/g)	ABTS (mgTE/g)	Phosphomolybdem (mmolTE/g)	CUPRAC (mgTE/g)	FRAP (mgTE/g)	MCA (mgEDTAE/g)
Halebi	17.22 ± 0.21^a^	3.82 ± 0.10^a^	33.93 ± 0.25^a^	52.52 ± 0.55^b^	0.60 ± 0.05^a^	48.07 ± 0.41^c^	26.01 ± 0.14^b^	17.68 ± 0.76^a^
Tekin	13.32 ± 0.55^a^	3.07 ± 0.17^a^	16.36 ± 0.35^a^	40.52 ± 0.38^b^	0.48 ± 0.005^a^	27.40 ± 0.18^c^	14.02 ± 0.60^b^	16.92 ± 1.54^a^
Uzun	24.61 ± 0.37^a^	2.57 ± 0.17^a^	47.87 ± 0.01^a^	85.80 ± 0.34^b^	0.78 ± 0.06^a^	85.54 ± 0.11^c^	42.96 ± 2.24^b^	19.14 ± 0.66^a^

*Note:* Three parallel means ± standard deviations are used to present the results. Statistically significant changes in the tested specimen (*p* < 0.05) are indicated by different letters.

Abbreviations: EDTAE, EDTA equivalents; GAE, gallic acid equivalents; MCA, metal chelating activity; RE, rutin equivalents; TE, trolox equivalents; TFC, total flavonoid content; TPC, total phenolic content.

The variety with the highest total phenolic content was Uzun (24.61 mg GAE/g), and the variety with the highest total flavonoid content was Halebi (3.82 mg RE/g). It was determined that Uzun showed the highest activity in DPPH, ABTS, phosphomolybdenum, CUPRAC, FRAP, and MCA antioxidant methods. As expected, the varieties with the highest total phenolic content also showed the highest antioxidant activity results. Among the phytochemicals responsible for antioxidant activity, tannic acid (24.005 mg/g) was determined to have the highest concentration in Uzun varieties. Thus, it can be said that LC–MS/MS results and antioxidant properties are compatible with each other. The results showed that the antioxidant property may be due to tannic acid. Foods with antioxidant properties are becoming increasingly popular. Therefore, three types of nuts have been shown to be important foods due to their antioxidant properties.

Antioxidants play very important roles in living systems. By removing free radicals, they prevent tissue and cell damage and eliminate oxidative stress. They are pioneers in the treatment of many diseases. They also remove oxidative deterioration in drugs and foods and show a protective effect against pathological conditions caused by oxidative stress in the body (İzol et al. 2024; Gulcin 2020).

In the study with *P. vera* L. cv. Siirt turpentine, antioxidant activities were determined by total phenolic and flavonoid content, ABTS, DPPH, CUPRAC, and FRAP methods. 53 phytochemicals were screened by the method in this study. The major components of the ethanol–water (50:50 v/v) extract of Siirt turpentine were protocatechuic acid and naringenin; the total phenolic and flavonoid contents were 38.852 mg QE/g and 12.5 mg GAE/g, respectively; the CUPRAC result was 0.098 μg/mL, the FRAP result was 0.328 μg/mL; the DPPH result was 346.6 (IC_50_) μg/mL; and the ABTS result was 231.1 (IC_50_) μg/mL (Karageçili, Yilmaz, et al. 2023). In this study, total phenolic and flavonoid contents were found to be in the ranges of 17–24 mg GAE/g and 2–3 mg RE/g, respectively, and were lower than the turpentine variety. While the antioxidant activities of the turpentine variety were low, the varieties in this study were found to be higher.

11 different pistachio varieties were analyzed for their antioxidant activity using total phenolic content, ABTS and DPPH tests. While there were statistical differences in the antioxidant properties of 11 different varieties, the study found that there were species with high antioxidant properties. In addition, the antioxidant properties of 11 varieties were evaluated among themselves. In the literature review, it was found that the shell part of the pistachio is generally studied, and the core part is less studied (Moreno‐Rojas et al. 2022).

In a study conducted in Türkiye, the antioxidant properties of the hull samples of 3 varieties of *Pistacia vera* L. were determined by the DPPH method in different extraction methods. As a result, it was determined that 3 varieties showed antioxidant properties (Karaoğlu and Tarhan 2022). In general, it has been reported in studies that the hull and kernel parts of pistachios show antioxidant properties.

### 3.3 Enzyme Inhibition Results

The results of AChE, BChE, tyrosinase, α‐glycosidase, and α‐amylase enzyme inhibitory activity of *P. vera* varieties are given in Table 3.

**TABLE 3:** AChE, BChE, tyrosinase, α‐glycosidase, and α‐amylase enzyme inhibitory activities results of *P. vera* varieties.

Samples	Enzyme inhibitory activities				
	AChE (mgGALAE/g)	BChE (mgGALAE/g)	Tyrosinase (mgKAE/g)	α‐Amylase (mmolACAE/g)	α‐Glucosidase (mmolACAE/g)
Halebi	2.06 ± 0.03^a^	2.13 ± 0.106^a^	30.03 ± 1.134^b^	0.67 ± 0.030^a^	2.16 ± 0.051^a^
Tekin	2.08 ± 0.04^a^	2.49 ± 0.251^a^	24.42 ± 1.429^b^	0.64 ± 0.001^a^	1.61 ± 0.029^a^
Uzun	2.25 ± 0.03^a^	2.54 ± 0.271^a^	43.82 ± 0.643^b^	0.69 ± 0.015^a^	2.94 ± 0.005^a^

*Note:* Three parallel means ± standard deviations are used to present the results. Statistically significant changes in the tested specimen (*p* < 0.05) are indicated by different letters.

Abbreviations: ACAE, acarbose equivalents; GALAE, galantamine equivalents; KAE, kojic acid equivalents.

The positive impact of consumed foods on human health is a sought‐after condition today. The potential therapeutic effect of three types of nuts against significant metabolic diseases will have a positive impact on their consumption. The anticholinergic and antidiabetic effects of these three types of nuts, in particular, have a positive impact on their consumption as food.

Since they can be used as medications to treat a variety of illnesses, such as diabetes, obesity, high blood pressure, and cancer, enzyme inhibitors play a significant role in the pharmaceutical business. The symptoms of many illnesses can be lessened by blocking certain enzymes. For instance, the rise in blood sugar levels in diabetics can be managed by blocking glucosidase and amylase (Yilmaz et al. 2024; İzol and Turhan 2024). Tyrosinase, for instance, is the primary enzyme involved in the manufacture of melanin; by inhibiting it, hyperpigmentation issues can be managed and numerous skin conditions can be treated.

In the study conducted in 2023, AChE and α‐glucosidase enzyme inhibitions of the Siirt Turpentine variety were determined. AChE and α‐glucosidase enzyme inhibitions were determined as 3.59 (IC_50_) μg/mL and 2.04 (IC_50_) μg/mL, respectively (Karageçili, Yilmaz, et al. 2023). In this study, AChE inhibition was 2.06–2.25 mg GALAE/g, and α‐glucosidase inhibition was in the range of 1.61–2.94. Enzyme inhibition was found to be high in both studies. Thus, it was determined that *P. vera* varieties are significant for Alzheimer's disease and diabetes.

In the study in which antidiabetic and anticholinesterase properties of pistachio fruit stalk extracts were analyzed, it was determined that different solvent extracts (ethanol, hexane, and chloroform) showed antidiabetic properties by inhibiting α‐amylase and α‐glycosidase enzymes. In fact, the inhibitory activity against the α‐glycosidase enzyme was higher than that of the antidiabetic drug acarbose standard. In addition, the anticholinesterase properties of the extracts were tested by AChE and BChE enzyme inhibition, and it was reported that all extracts showed inhibitory properties. It was also reported that cholinesterase inhibition was lower against the standard drugs neostigmine and galantamine (Lawali et al. 2020).

### 3.4 Molecular Docking Result

Generally speaking, molecular docking calculations are carried out to discover molecules' active sites and to support experimental operations. Using molecular docking calculations, molecular modeling is a crucial technique for researching how chemicals interact with proteins (Yildirim, Kilic, et al. 2025). This technique establishes the contact between molecules and proteins as well as the activity of molecules against them; the more interaction there is, the more active the molecules are. Numerous parameters were computed as a result of the calculations, and each one provides details about various molecular characteristics (Necip 2024; Yildirim, Dogan, et al. 2025). Upon analyzing these factors, the docking score parameter is the first one that establishes the molecules' activity.

We can say that the lower the docking score value, the stronger the connection (Dikme et al. 2024). The values between catechin, epicatechin gallate, epigallocatechin gallate, gallic acid and isoquercitrin with docking score values of 2Y9X, 6GXV, 5NN8, 6EQP and 4EY7 proteins are given in Table 4.

**TABLE 4:** Catechine, epicatechin gallate, epigallocatechin gallate, gallic acid, and isoquercitrin with docking score values of 2Y9X, 6GXV, 5NN8, 6EQP, and 4EY7 proteins.

Molecule/Protein	Docking score values (kcal/mol)				
	2Y9X	6GXV	5NN8	6EQP	4EY7
Catechine	−5.565	−6.569	−6.154	−7.082	−8.892
Epicatechin gallate	−6.322	−5.514	−4.947	−4.987	−4.895
Epigallocatechin gallate	−6.192	−5.955	−6.349	−6.547	−6.604
Gallic acid	−8.191	−5.821	−5.259	−7.706	−6.884
Isoquercitrin	−6.033	−4.977	−5.392	−6.436	−6.738

**FIGURE 3:**
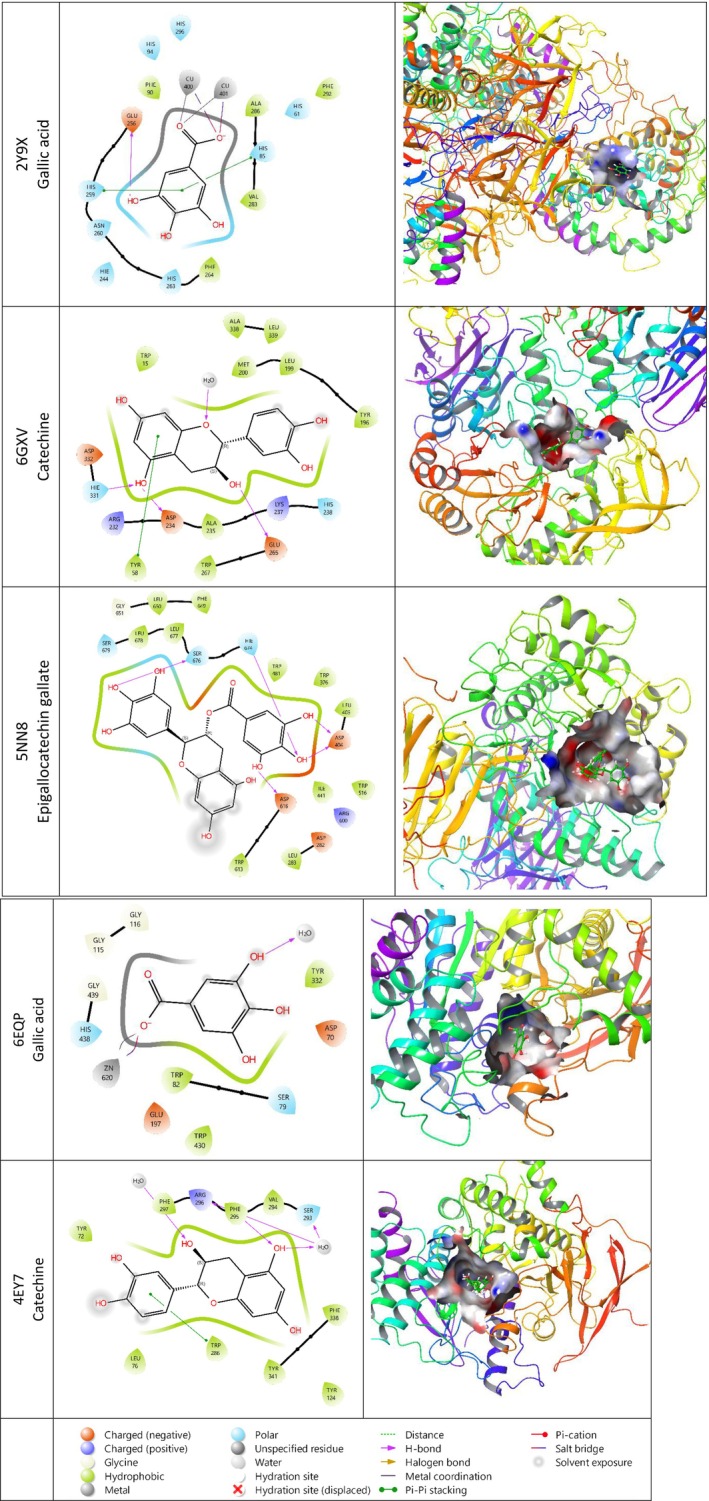
Protein–ligand interaction 2D and 3D.

In this study, the binding affinities of various bioactive compounds (catechin, epicatechin gallate, epigallocatechin gallate, gallic acid and isoquercitrin) with different protein targets (2Y9X, 6GXV, 5NN8, 6EQP, and 4EY7) were examined using molecular docking analysis. Docking scores were calculated in terms of binding free energy (kcal/mol), with negative values indicating higher binding affinity. According to the results, among the compounds examined, gallic acid has the highest binding affinity, especially with 2Y9X (−8.191 kcal/mol) and 6EQP (−7.706 kcal/mol) proteins, indicating that gallic acid can bind strongly to the active sites of the relevant proteins. It shows. Catechin reached the highest binding score of −8.892 kcal/mol with the 4EY7 protein. Among other compounds, epigallocatechin gallate showed overall moderate binding affinity. These findings may reveal the interaction potential of the examined compounds with certain proteins, allowing further investigation of the relevant biological mechanisms. In particular, we can say that gallic acid and catechin play a role in the inhibition effects because they show high affinity with certain protein targets. Protein–ligand interaction in 2D and 3D, shown in Figure 3.

Catechin interacted by binding to the active site of the 6GXV protein. Hydrogen bonding with Glu 265, Asp 234, and HIE 331 indicates that catechin binds to a hydrophilic region. The presence of negatively charged amino acids such as Asp 234 and Glu 265 suggests that the ligand has electrostatic compatibility with this region.

Epigallocatechin gallate formed hydrogen bonds and electrostatic interactions by binding to the active site of the 5NN8 protein. The region where epigallocatechin gallate binds is hydrophilic and contains negatively charged amino acids, which may contribute to the strong binding of the ligand.

Catechin bound to the active site of the 4EY7 protein and formed various hydrogen bonds and pi–pi interactions. The binding site consists of hydrophilic and aromatic amino acids and contributes to the stable binding of catechin. Glu 265, negatively charged, can be a hydrogen bond acceptor or donor. Asp 234 is a negatively charged amino acid and can contribute to electrostatic interactions. HIE 331 (Histidine): Depending on pH, it can be protonated and make hydrogen bonds. Tyr 58 (Tyrosine) may be a pi–pi stacking interaction between the aromatic ring and the aromatic groups of catechin. Trp 286 (Tryptophan) is a hydrophobic and aromatic amino acid and can increase the stability of the ligand.

The interaction of catechin with the 4EY7 protein may be more stable than with 6GXV because pi–pi stacking interactions provide an additional contribution. It is also supported by docking score values.

The presence of various groups in catechin, epicatechin gallate, epigallocatechin gallate, gallic acid, and isoquercitrin may also improve their activity by altering their physicochemical characteristics and pharmacokinetic parameters to improve their bioavailability, metabolic stability, and receptor‐binding affinity, according to in vitro and in silico results.

**FIGURE 4:**
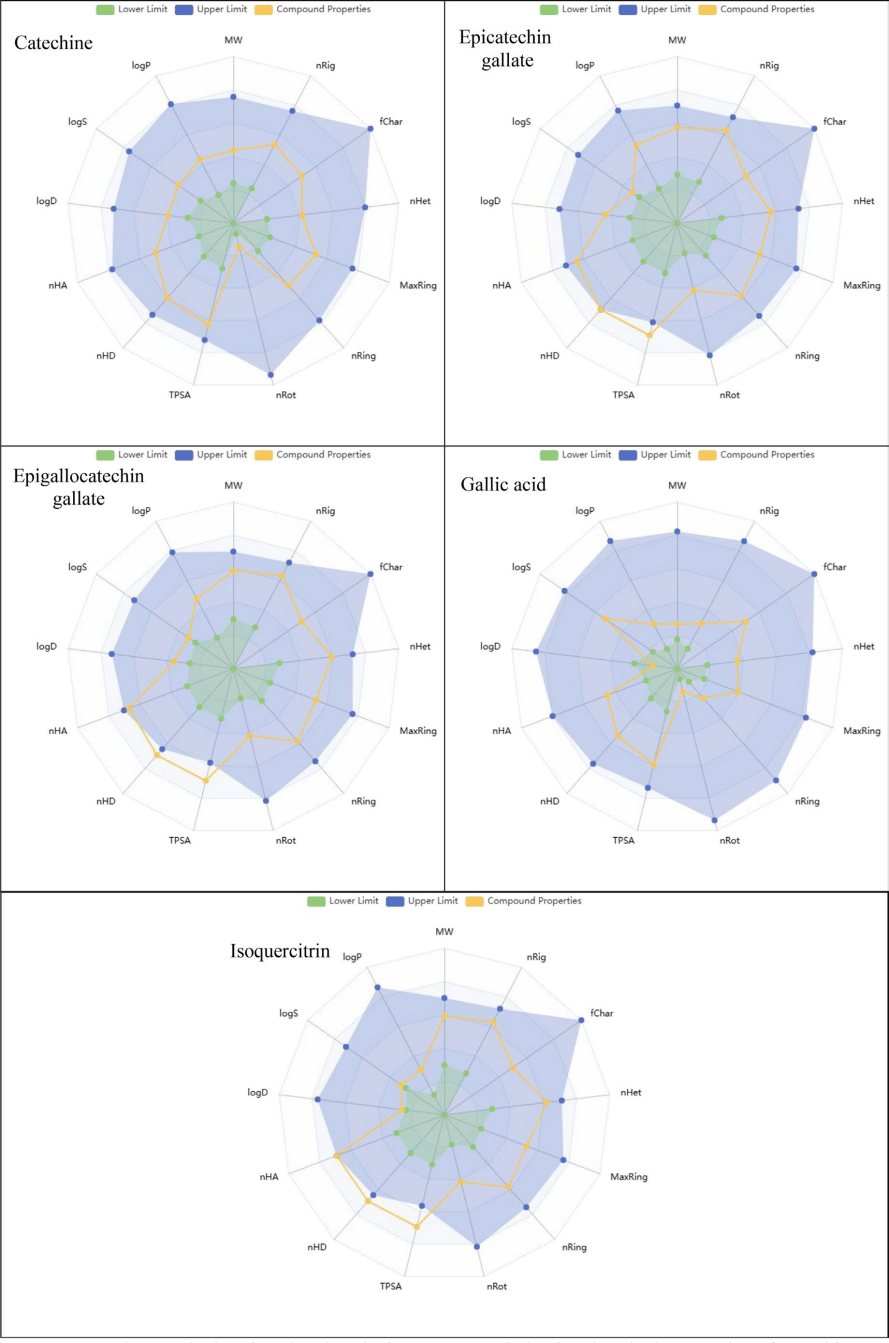
Radar graph showing the chemical structure and physicochemical properties of catechine, epicatechin gallate, epigallocatechin gallate, gallic acid, and isoquercitrin.

The effects and reactions of these investigated compounds in human metabolism were investigated using ADME/T analysis (absorption, distribution, metabolism, excretion, and toxicity). This analysis was used to determine the compounds' absorption by human metabolism, distribution within the metabolism, excretion from the metabolism, and, ultimately, toxicity levels within the metabolism. Many parameters that analyze the chemical properties of molecules are calculated, such as mol_MW (molar mass of molecules), Molecular Weight (MW), Volume (molecular volume), LogP (the degree of lipophilicity of the molecule), TPSA (Total Polar Surface Area, refers to the polar surface area of the molecule, affects bioavailability), nRot (number of rotationally free bonds), LogS (degree of water solubility), and nHA and nHD (refers to the number of atoms that accept and give hydrogen bonds). The physicochemical and ADME properties of catechin, epicatechin gallate, epigallocatechin gallate, gallic acid, and isoquercitrin are given in Table 5. The radar graphs of these compounds are shown in Figure 4.

**TABLE 5:** Physicochemical and ADME properties of catechine, epicatechin gallate, epigallocatechin gallate, gallic acid and isoquercitrin.

	Catechine	Epicatechin gallate	Epigallocatechin gallate	Gallic acid	Isoquercitrin	Optimal
Molecular weight (MW)	290.08	442.09	450.08	170.02	464.1	100–600
Volume	279.249	416.38	425.17	154.477	421.937	
Density	1.039	1.062	1.077	1.011	1.1	
nHA	6.0	10.0	11	5	12	0–12
nHD	5.0	7.0	8	4	8	0–7
nRot	1.0	4.0	4	1	4	0–11
nRing	3.0	4	4	1	4	0–6
MaxRing	10.0	10	10	6	10	0–18
nHet	6.0	10	11	5	12	1–15
fChar	0.0	0	0	0	0	−1
nRig	17.0	24	24	7	24	0–30
Flexibility	0.059	0.167	0.167	0.143	0.167	
Stereo centers	2.0	2	2	0	5	< 2
TPSA	110.38	177.14	197.37	97.99	210.51	0–140
logS	−2.581	−3.493	−3.483	−1.55	−3.63	
logP	1.173	1.672	1.372	0.692	0.697	0–3
logD7.4	1.537	1.693	1.4	0.615	1.087	1–3
Medicinal chemistry						
Lipinski rule	**	**	*	**	*	
Pfizer rule	**	**	**	**	**	
GSK rule	**	*	*	**	*	

*Note:* *, Rejected; **, Accepted.

The molecular weights of all compounds are in the range of 100–600 Da, within the accepted limits for drug‐like molecules. Although the number of atoms capable of hydrogen bonding generally complies with Lipinski's rules, it exceeds the limit values in some compounds. In particular, isoquercitrin (nHA = 12, nHD = 8) may violate Lipinski's rules due to its high hydrogen bonding capacity.

TPSA, which is an important parameter in terms of bioavailability of drugs, should be below 140 Å^2^. However, epicatechin gallate (177.14 Å^2^), epigallocatechin gallate (197.37 Å^2^) and isoquercitrin (210.51 Å^2^) exceed this limit, suggesting that their bioavailability through passive diffusion may be low. The log_*P*_ values of the compounds are in the range of 0–3 and are at acceptable levels in terms of drug design. This indicates that the lipophilic properties of the compounds are sufficient and membrane permeability may be appropriate. Although the water solubility (log_S_) of the compounds is relatively low for epicatechin gallate (−3.493), epigallocatechin gallate (−3.483), and isoquercitrin (−3.63), it is critical in terms of bioavailability because it does not reach a certain level. While gallic acid and catechin offer more advantageous properties in terms of pharmacokinetics, it is considered that the bioavailability of compounds such as isoquercitrin and epigallocatechin gallate may be limited due to the high number of TPSA and hydrogen bond‐forming atoms.

## 4 Conclusion

Pistachios are used in many foods and are very important foods. Particular value is attached to the varieties grown in Türkiye. Determining the chemical composition and biological activity of the pistachio kernel portion is essential. In this study, the phytochemical profile, antioxidant, anticholinergic, and antidiabetic properties, and molecular docking study of three varieties of pistachios from Türkiye were comprehensively analyzed. In the literature, it was observed that the kernel part, which is the consumed part of these three varieties, has not been studied in this context, and for this reason, this study was determined to be the first. It is foreseen that three varieties of pistachios can be used in diabetes, Alzheimer's, skin diseases, and antioxidant‐effective drugs and can take part in the treatment processes of patients. The ability to optimize the design and development of new compounds that interact with specific proteins is made feasible by molecular docking calculations and methodologies, which are becoming essential tools in contemporary drug discovery procedures. However, in vivo and then clinical studies are needed.

This study has determined that these three pistachios have significant effects on human health and are significant in terms of food consumption, as foods are currently expected to have a high positive impact on human health.

## Author Contributions

E.İ. planned and wrote the study. E.İ., B.B., and M.T. collected the samples and performed the extraction. E.İ., B.B., M.A.Y., Ş.K., O.Ç., and G.Z. performed the analyses. A.N. conducted docking studies.

## Funding

This work was supported by Turkiye Bilimsel ve Teknolojik Araştırma Kurumu.

## Conflicts of Interest

The authors declare no conflicts of interest.

## Data Availability

The data that support the findings of this study are available on request from the corresponding author.
